# Lupus Enteritis as an Initial Presentation of Systemic Lupus Erythematosus

**DOI:** 10.1155/2014/962735

**Published:** 2014-09-11

**Authors:** Sisira Sran, Manpreet Sran, Narmisha Patel, Prachi Anand

**Affiliations:** ^1^Department of Medicine, Nassau University Medical Center, 2201 Hempstead Turnpike, East Meadow, NY 11554, USA; ^2^Department of Rheumatology, Nassau University Medical Center, 2201 Hempstead Turnpike, East Meadow, NY 11554, USA

## Abstract

Systemic lupus erythematosus (SLE) is an autoimmune disorder which can affect multiple organs and clinical presentation is often a myriad of symptoms; therefore, the index of suspicion should rise when evaluating patients with multiorgan symptomatology. Lupus enteritis is a distinct subset of SLE, defined as either vasculitis or inflammation of the small bowel, with supportive image and/or biopsy findings. The clinical picture of lupus enteritis is often nonspecific, with mild to severe abdominal pain, diarrhea, and vomiting being the cardinal manifestations. Although considered a form of visceral or serosal vasculitis, lupus enteritis is seldom confirmed on histology, making computerized tomography (CT) the gold standard for diagnosis. Lupus enteritis is generally steroid-responsive, and the route of administration is based on clinical status and organ involvement, with preference for intravenous (IV) route in flares with significant tissue edema. The following case describes a young woman presenting with lupus enteritis and lupus panniculitis as an initial manifestation of SLE, the utilization of abdominal CT in diagnosis, and current treatment protocols used for lupus enteritis.

## 1. Introduction

SLE generally affects young to middle aged women, commonly presenting as a triad of fever, rash, and joint pain. However, SLE can present in a complex fashion, varying based on the degree and severity of organ involvement. Gastrointestinal symptoms are common in SLE, and more than half of the conditions are caused by adverse reactions to medications, viral or bacterial infections [1]. Other causes include lupus mesenteric vasculitis, which can lead to protein-losing enteropathy, intestinal pseudoobstruction, acute pancreatitis, and other rare complications, such as celiac disease and inflammatory bowel diseases. Abdominal pain in patients with SLE may also reflect underlying vasculitis and thrombosis, which can lead to life-threatening ischemia and perforation, if not promptly treated. The following case describes a young woman presenting with lupus enteritis and lupus panniculitis as the initial manifestation of SLE, the importance of early disease recognition, utilities of abdominal CT in diagnosis, and current treatment protocols for lupus enteritis.

## 2. Case Presentation

**Figure 1:**
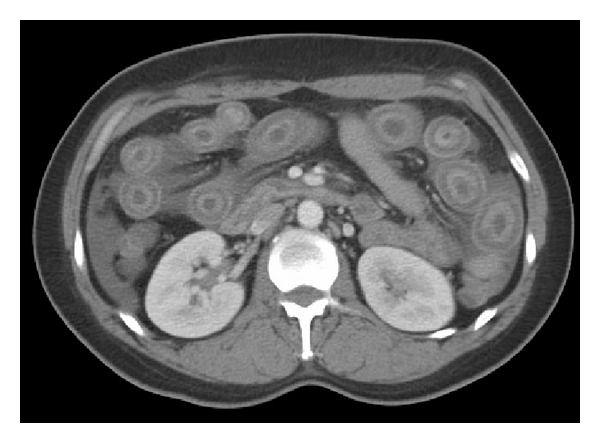
Computed tomography of the abdomen illustrating bowel loops with edema also referred to as target sign.

A 34-year-old Hispanic female with no significant medical history arrived at the emergency department with 3 days of abdominal pain, vomiting, and painful lesions on the skin of the lower extremities. The patient described the pain as diffuse, constant, and dull in intensity, without any alleviating factors or associated symptoms. On physical examination, vitals were within normal limits. The abdomen was diffusely tender and the lower extremities revealed multiple crops, nontender 1-2 cm subcutaneous nodules, without evidence of induration or suppuration. Laboratory tests revealed microcytic anemia (hemoglobin of 9.8 g/dL), a positive antinuclear antibody titer of 1 : 180, low complement levels, negative serology for anti-DsDNA, anti-RNP, anti-Smith, anti-SSA, and anti-SSB antibodies, and undetectable cryoglobulins levels. CT of the abdomen revealed diffuse edema and thickening of the small bowel wall with a moderate amount of abdominopelvic ascites, primarily surrounding loops of small bowel, the liver, and spleen and in the pelvic cavity (Figure 1). The patient was admitted and given intravenous hydration, ciprofloxacin, and metronidazole for presumed infectious enteritis; however, the following day the symptoms did not improve. An upper endoscopy did not reveal any significant abnormalities, and biopsies along with mesenteric angiography were negative for vasculitis or ischemia. A skin biopsy of the nodular lesions was then performed which revealed lobular and septal panniculitis, with inflammation primarily composed of neutrophils without any granulomas. The patient was given systemic steroids and within a few days the abdominal pain and skin lesions began to resolve. The patient was discharged on hydroxychloroquine and a tapering dose of steroids.

## 3. Discussion

The clinical picture of lupus enteritis is often nonspecific, with abdominal pain, diarrhea, and vomiting being the cardinal manifestations with jejunum (80%) or with ileum (85%) involvement [2]. The pathogenesis is unclear but has been attributed to immune-complex deposition and complement activation, with subsequent submucosal edema [3]. Although considered a form of visceral or serosal vasculitis, lupus enteritis is seldom confirmed on histology, making computerized tomography the gold standard for diagnosis. There are three classic patterns suggestive of lupus enteritis: (1) bowel wall thickening greater than 3 mm, also referred to as target sign, (2) engorgement of the mesenteric vessels (coombs sign), and (3) increased attenuation of mesenteric fat [2] (Figure 1). Arteriography may reveal arterial narrowing and distended loops of bowel [4]. One should always consider mesenteric vasculitis in the differential diagnosis as overlooking the diagnosis can have grave consequences. One autopsy study found that 60–70% of SLE patients had evidence of peritonitis, whereas only around 10% of them were recognized clinically [5]. Signs of perforation may be subtle and masked in patients taking steroids; therefore, abdominal pain in a lupus patient must be addressed and evaluated. Lin et al. suggested that SLE should be suspected in any patient with CT findings of enteral vasculitis or ischemic enteritis, even without lupus-related symptoms or signs and complement levels [6]. C3/C4 levels may be helpful in the differential diagnosis.

Steroids are generally considered to be first line therapy for lupus enteritis. Steroid administration may be IV or by mouth based on clinical status or other organ involvement, with preference for IV in case of severe lupus flare because of potentially reduced drug absorption from tissue edema due to enteritis [7]. In steroid resistant cases, oral mycophenolate may be another option [8]. There has been one single report of a patient who responded to the EURO lupus cyclophosphamide regimen [3]. Even if patients respond initially to steroids, there is a high predilection for recurrence. A predictor of risk of recurrence for lupus enteritis is bowel wall thickness greater than 9 mm and the recurrence rate of lupus enteritis correlates with a lower cumulative dosage of prednisolone and a shorter duration of treatment [3, 9].

## 4. Conclusion

Lupus enteritis underlies a broad spectrum of processes which includes mesenteric arteritis, intestinal vasculitis, lupus peritonitis, and abdominal serositis. Patients who have complaints of abdominal pain should be evaluated carefully as overlooking the diagnosis and delaying treatment can result in bowel ischemia and perforation. The diagnosis of lupus enteritis is based on classical CT findings (bowel wall edema with target sign, mesenteric abnormalities, and ascites) as histopathology seldom confirms the diagnosis. Typically, lupus enteritis is steroid-responsive with an overall excellent prognosis and immunosuppressive treatment is reserved for recurrent enteritis or severe SLE cases with multiorgan involvement. This case illustrates that SLE involves various systems and can present in a multitude of ways, making it important for clinicians to consider the differential diagnosis, especially in a young woman with complex symptomatology.
